# Supplementary material for: Collaborative intelligence in AI: Evaluating the performance of a council of AIs on the USMLE
Source: PLOS Digit Health. 2025 Oct 9;4(10):e0000787. doi: 10.1371/journal.pdig.0000787 (PMC12510544; doi:10.1371/journal.pdig.0000787)
Supplement: S2 File — (ZIP) [file pdig.0000787.s002.zip › S2 Data Raw data files containing unprocessed question inputs and LLM outputs including transcripts of deliberations/STEP3_Question-68-1_Type-MC-NJ.docx]

**Facilitator** **[Round 1]:** A 19-year-old woman comes to her university's health center because of a 1-week history of nasal stuffiness, occasional clear rhinorrhea, and a mild sore throat. Two days ago, she began to feel hot, but she has not taken her temperature. Medical history is unremarkable and her only medication is an oral contraceptive. She has no history of allergies. She does not smoke cigarettes. BMI is 22 kg/m². Vital signs are temperature 38.1°C (100.6°F), pulse 88/min, respirations 16/min, and blood pressure 116/74 mm Hg. Physical examination discloses tenderness over the left maxillary sinus without purulent drainage. There is clear fluid behind the left tympanic membrane, enlarged tonsils bilaterally, and pain with tapping of the left upper incisors. The left frontal sinus does not transilluminate. Cardiopulmonary examination discloses no abnormalities. Which of the following is the most likely underlying mechanism of this patient's sinusitis? (A) Eustachian tube dysfunction (B) Mucosal edema (C) Nasal polyps (D) Oral contraceptive use (E) Tonsillar hyperplasia

**AI_C:** 1) RESTATING THE QUESTION: The question asks what is the most probable cause of sinusitis in a 19-year-old woman who has presented with nasal stuffiness, clear rhinorrhea, a mild sore throat, fever, and symptoms suggestive of sinusitis such as a tenderness over the maxillary sinus, fluid behind the tympanic membrane, and enlarged tonsils. 2) PLANNING: The patient's symptoms could be attributed to a number of underlying etiologies, including Eustachian tube dysfunction, mucosal edema, nasal polyps, use of oral contraceptives, or tonsillar hyperplasia. However, the question requires a singular most likely cause, which necessitates a closer examination of each of these possibilities. 3) CLINICAL REASONING: a) Eustachian tube dysfunction could cause fluid accumulation behind the tympanic membrane, but it would not typically result in symptoms of sinusitis. The Eustachian tube connects the middle ear to the nasopharynx and its dysfunction can cause conditions like otitis media, but not sinusitis. b) Mucosal edema can be a cause of sinusitis. The inflammation of the mucosa leads to blockage of sinus ostia leading to accumulation of secretions and bacterial growth. c) Nasal polyps are typically not painful and are associated with a history of chronic sinusitis, allergies, or asthma, none of which this patient has. d) Oral contraceptives can cause changes in mucus, but are more commonly associated with cervical mucus alterations rather than nasal or sinus mucosa. e) Tonsillar hyperplasia, while present, generally doesn't cause sinusitis. Rather, it's a consequence of a chronic infection itself, and this patient doesn't display symptoms of a chronic infection. 4) CONCLUDING: Based on these clinical thoughts, the most likely underlying mechanism of this patient's sinusitis is mucosal edema. It's a common cause of sinusitis and is consistent with her symptoms and signs. 5) RESPONSE: Therefore, the most likely underlying cause of this patient's sinusitis is mucosal edema (option B), arising from inflammation of the nasal and sinus mucosa, leading to blockage of sinus ostia and thereby accumulation of secretions and bacterial overgrowth.

**AI_E:** (1) RESTATING THE QUESTION: We are trying to pinpoint the main foundational factor leading to the sinusitis of this young woman. In the medical context, sinusitis refers to an inflammation or swelling of the tissue lining the sinuses, often resulting in blocked sinuses filled with fluid and germs that can cause infection. The possible underlying causes in this case are eustachian tube dysfunction, mucosal edema, nasal polyps, oral contraceptive use, and tonsillar hyperplasia. Eustachian tube dysfunction refers to problems with the tube that links the middle ear to the pharynx. Mucosal edema signifies swelling in the mucous membrane, nasal polyps are a type of noncancerous tumor that grows within the lining of the nose or sinuses. Oral contraceptive use points to her regimen of birth control pills, while tonsillar hyperplasia refers to an abnormal enlargement of the tonsils. (2) PLANNING: We can assume that her sinusitis is due to either an anatomical abnormality or an inflammatory response. Nasal polyps and tonsillar hyperplasia are both examples of anatomical abnormalities. However, her exam lacks the commonly seen symptoms such as notable obstruction of nasal breathing or snoring. Eustachian tube dysfunction, on the other hand, frequently appears with ear symptoms, which is not prominent in this case. Thus, these options seem less likely. The other potential causes relate to an inflammatory response: mucosal edema and consequence of oral contraceptive use. (3) CLINICAL REASONING: Given this patient's symptoms of nasal stuffiness, clear rhinorrhea, sore throat, and fever–all hallmark symptoms of viral upper respiratory infections, we can infer that inflammation is indeed taking place. This inflammation can lead to the blockage of the ostiomeatal complex (OMC), a common pathway shared by the frontal, maxillary, and anterior ethmoid sinuses, and contributes to the development of sinusitis. This blockage is typically caused by mucosal edema rather than the oral contraceptives. While contraceptives can cause mucosal changes in some areas of the body, it's not typically associated with sinusitis. (4) CONCLUDING: Considering the fact that this patient's symptoms are consistent with a viral respiratory infection, combined with the fact that her physical exam reveals inflammation but no evidence of anatomical abnormalities or otitis media, it seems most likely that her sinusitis resulted from mucosal edema. (5) RESPONSE: The most likely underlying mechanism of this patient's sinusitis is mucosal edema (B). This is probably due to swelling and inflammation caused by a viral infection leading to blockage of the ostiomeatal complex and subsequently, sinusitis.

**AI_D:** 1) RESTATING THE QUESTION: A 19-year-old female student, without significant past medical history, has symptoms of sinusitis namely nasal congestion, clear rhinorrhea, mild pharyngeal discomfort, and fever. She is currently on hormonal birth control medication, is non-allergic, and non-smoker. Her BMI and the rest of her vital signs seem to be within the normal range. On examination, she has tenderness over the left maxillary sinus, enlarged tonsils, and otic fluid, without any obvious cardiorespiratory findings. The question asks about the most likely cause of these sinusitis symptoms. 2) PLANNING: The five possible causes of sinusitis listed in the question include Eustachian tube dysfunction (a blockage of the narrow channel connecting the middle ear to the back of the nose), mucosal edema (swelling of the membrane lining the sinuses), nasal polyps (small, benign growths in the lining of the nose or sinuses), oral contraceptive use (which can alter immunological and vascular responses), and tonsillar hyperplasia (enlargement of the tonsils). It's important to assess how each of these factors could contribute to the symptoms seen in the patient to understand the most likely cause of her sinusitis. 3) CLINICAL REASONING: While Eustachian tube dysfunction could explain the clear fluid behind the tympanic membrane, it doesn't usually cause sinus tenderness and is often associated with aural symptoms which are not highlighted in this case. Nasal polyps and tonsillar hyperplasia may obstruct sinus drainage, leading to sinusitis, but they usually cause chronic, not acute, symptoms. Oral contraceptive use could arguably affect immune responses, making the patient more susceptible to infections, but it doesn't directly contribute to sinusitis and doesn't explain the localized physical findings observed. Considering the acuity of the symptoms and the localised findings of tenderness over the left maxillary sinus and non-transilluminating left frontal sinus, mucosal edema seems to be the most likely underlying mechanism. Mucosal edema would trap fluid in the sinuses, impairing sinus drainage, and thus create a favored environment for viral replication (most commonly rhinoviruses) and secondary bacterial infections (especially Streptococcus pneumoniae, Haemophilus influenzae, and Moraxella catarrhalis). 4) CONCLUDING: Given this context and based on my clinical reasoning, I would conclude that the most likely underlying mechanism behind this patient's sinusitis is mucosal edema. 5) RESPONSE: (B) Mucosal edema is probably the main mechanism causing this patient's sinusitis.

**AI_B:** (1) RESTATING THE QUESTION: To paraphrase, we are asked to identify the most probable cause underlying the symptoms of sinusitis in this 19-year-old woman. These symptoms include nasal congestion, clear rhinorrhea, mild throat discomfort, fever, and tenderness and inability to transilluminate the left maxillary and frontal sinuses. Options provided include Eustachian tube dysfunction, mucosal edema, nasal polyps, oral contraceptive use, and enlarged tonsils. (2) PLANNING: To detect the most possible cause, we must consider each assumption. Eustachian tube dysfunction can cause fluid accumulation in the middle ear, although it is more associated with otitis media than sinusitis. Mucosal edema usually occurs in response to various triggers, such as respiratory infections or allergies, resulting in sinus drainage impairment. Nasal polyps could obstruct sinus drainage, but they usually lead to chronic sinusitis, not the acute presentation this patient has. Oral contraceptives might theoretically influence the mucosal system through hormonal changes, but they're not a well-known cause of sinusitis. Finally, tonsillar hyperplasia could potentially obstruct drainage, but again, this isn't the typical manifestation. (3) CLINICAL REASONING: Starting with Eustachian tube dysfunction, we see that, while it could be part of the picture, this is mainly associated with otitis media and wouldn't principally lead to the acute sinusitis the patient is experiencing. With nasal polyps, these typically produce chronic symptoms and would likely have a different presentation than what this patient is experiencing, ruling this out. This patient does not present with any symptoms suggesting oral contraceptive-induced sinusitis. Indeed, to date, there's no documentation linking oral contraceptive use to the development of sinusitis. His universal symptom of sinusitis could have been exacerbated by tonsillar hyperplasia, but this kind of mechanical obstruction would likely lead to chronic sinusitis as opposed to an acute condition. Mucosal edema, however, could be a potential cause here. Sinusitis typically occurs after an upper respiratory infection, which can cause inflammation and congestion of the nasal and sinus mucosa. This inflammation can cause edema and obstruction of the sinus ostia, leading to impaired sinus drainage and secondary sinusitis. (4) CONCLUDING: After going step by step and ruling out the less probable causes, the most commonplace basis for acute sinusitis, like presented in this case, is mucosal edema. This condition usually occurs following upper respiratory infections or stimulation of the immune system and results in inflammation and swelling of the nasal and sinus mucosa. (5) RESPONSE: Therefore, the most likely underlying mechanism of this patient's sinusitis is (B) Mucosal edema.

**AI_A:** (1) RESTATING THE QUESTION: The query is trying to find out the essential cause behind the sinusitis experienced by a 19-year-old female patient, who has been suffering from nasal stuffiness, occasionally clear rhinorrhea and a mild sore throat for a whole week. She started feeling feverish two days ago. Based on her medical history, she is generally healthy and she is only on one drug; an oral contraceptive. She does not have any allergies, does not smoke and her BMI is normal. Following an examination, tenderness was found over her left maxillary sinus but without any purulent discharge. Clear fluid was found behind her left tympanic membrane. Her tonsils are enlarged and there is pain when her left upper incisors are tapped. There was lack of transillumination in her left frontal sinus. Her cardiopulmonary examination reveals no irregularities. Sinusitis is an inflammation or swelling of the tissue lining the sinuses, typically caused by a virus but can be due to bacteria or fungi. (2) PLANNING: The possible underlying mechanisms mentioned in the question are Eustachian tube dysfunction, mucosal edema, nasal polyps, oral contraceptive use, and tonsillar hyperplasia. Evaluating each, Eustachian tube dysfunction could cause fluid backup in the middle ear, but the symptoms are mainly ear fullness, popping sounds or decreased hearing, not sinusitis. Nasal polyps are noncancerous growths in the sinus or nose lining that may cause sinusitis but are usually associated with chronic inflammation, and the patient's condition seems acute. Oral contraceptives could theoretically alter mucus production as a side effect, but this does not typically lead to sinusitis. Tonsillar hyperplasia could obstruct the airways and cause symptoms similar to sinusitis, but it won't actually cause the sinus inflammation. Mucosal edema, on the other hand, could block sinus drainage, leading to sinusitis, particularly in an acute setting like the patient's. (3) CLINICAL REASONING: Following the evaluation, Mucosal edema would seem to be the most likely cause. Upper respiratory infections can inflame and swell the sinus mucosa, blocking fluid drainage from the sinus cavities, which can result in fluid build-up and secondary bacterial infection - leading to sinusitis. This aligns with the patient's symptoms, particularly the tenderness over left maxillary sinus, the clear fluid behind the tympanic membrane, and lack of transillumination in her left frontal sinus, which are all suggestive of an acute sinusitis. (4) CONCLUDING: Taking into account the patient's age, symptomatology, and presentation, along with the typical pathogenesis of sinusitis, the underlying mechanism in this case would be mucosal edema as a result of an upper respiratory infection. Other options fail to align with the patient's presentation or would commonly result in other symptomatology. (5) RESPONSE: This patient's sinusitis is most likely caused by an underlying mechanism of Mucosal edema (option B).

**Facilitator** The consensus statement is as follows: The question seeks to identify the most probable cause of sinusitis in a 19-year-old woman presenting with symptoms such as nasal congestion, clear rhinorrhea, mild throat discomfort, fever, and signs suggestive of sinusitis. The potential causes provided include Eustachian tube dysfunction, mucosal edema, nasal polyps, oral contraceptive use, and tonsillar hyperplasia. Upon evaluating each potential cause, Eustachian tube dysfunction, while it could cause fluid accumulation in the middle ear, is more associated with otitis media than sinusitis. Nasal polyps and tonsillar hyperplasia could obstruct sinus drainage, leading to sinusitis, but they usually cause chronic, not acute, symptoms. Oral contraceptives might theoretically influence the mucosal system through hormonal changes, but they're not a well-known cause of sinusitis. Mucosal edema, however, emerges as the most likely cause. Sinusitis typically occurs after an upper respiratory infection, which can cause inflammation and congestion of the nasal and sinus mucosa. This inflammation can cause edema and obstruction of the sinus ostia, leading to impaired sinus drainage and secondary sinusitis. This aligns with the patient's symptoms, particularly the tenderness over the left maxillary sinus, the clear fluid behind the tympanic membrane, and lack of transillumination in her left frontal sinus, which are all suggestive of an acute sinusitis. Therefore, the consensus is that the most likely underlying mechanism of this patient's sinusitis is mucosal edema. This is probably due to swelling and inflammation caused by a viral infection leading to blockage of the ostiomeatal complex and subsequently, sinusitis.
